# Complete *DPYD* genotyping combined with dihydropyrimidine dehydrogenase phenotyping to prevent fluoropyrimidine toxicity: A retrospective study

**DOI:** 10.1002/cam4.7066

**Published:** 2024-03-25

**Authors:** Côme De Metz, Benjamin Hennart, Estelle Aymes, Pierre‐Yves Cren, Niels Martignène, Nicolas Penel, Maël Barthoulot, Aurélien Carnot

**Affiliations:** ^1^ Department of Medical Oncology Centre Oscar Lambret Lille France; ^2^ Toxicology Unit, Biology and Pathology Centre Lille University Medical Centre Lille France; ^3^ Department of Biostatistics Centre Oscar Lambret Lille France; ^4^ Univ. Lille, CHU Lille, ULR 2694 ‐ Metrics: Evaluation des technologies de santé et des pratiques médicales Lille France

**Keywords:** capecitabine, fluorouracil, genotype, high‐throughput nucleotide sequencing, neoplasms, phenotype

## Abstract

**Introduction:**

In April 2019, French authorities mandated dihydropyrimidine dehydrogenase (DPD) screening, specifically testing uracilemia, to mitigate the risk of toxicity associated with fluoropyrimidine‐based chemotherapy. However, this subject is still of debate as there is no consensus on a standardized DPD deficiency screening test. We conducted a real‐life retrospective study with the aim of assessing the impact of DPD screening on the occurrence of severe toxicity and exploring the potential benefits of complete genotyping using next‐generation sequencing.

**Methods:**

All adult patients consecutively treated with 5‐fluorouracil (5‐FU) or its oral prodrug at six cancer centers between March 2018 and February 2019 were considered for inclusion. Dihydropyrimidine dehydrogenase deficiency screening included gene encoding DPD (*DPYD)* genotyping using complete genome sequencing and DPD phenotyping (uracilemia or dihydrouracilemia/uracilemia ratio) or both tests. Associations between each DPD screening method and (i) severe (grade ≥3) early toxicity and (ii) fluoropyrimidine dose reduction in the second chemotherapy cycle were evaluated using multivariable logistic regression analysis. Furthermore, we assessed the concordance between DPD genotype and phenotype using Cohen's kappa.

**Results:**

A total of 551 patients were included. Most patients were tested for DPD deficiency (86%) including *DPYD* genotyping only (6%), DPD phenotyping only (8%), or both (72%). Complete DPD deficiency was not detected in the study population. Severe early toxicity events were observed in 73 patients (13%), with two patients (0.30%) presenting grade 5 toxicity. Despite the numerically higher toxicity rate in untested patients, the occurrence of severe toxicity was not significantly associated with the DPD screening method (*p* = 0.69). Concordance between the DPD genotype and phenotype was weak (Cohen's kappa of 0.14).

**Conclusion:**

Due to insufficient numbers, our study was not able to demonstrate any added value of *DPYD* genotyping using complete genome sequencing to prevent 5‐FU toxicity. The optimal strategy for DPD screening before fluoropyrimidine‐based chemotherapy requires further clinical evaluation.

## 1 INTRODUCTION

Fluoropyrimidine (FP)‐based chemotherapy regimens are widely used for metastatic tumors or as adjuvant therapy for many solid cancers, mainly gastrointestinal (oesogastric, pancreas, and colorectal cancers), breast, and head and neck cancers. Currently, FP includes 5‐fluorouracil (5‐FU) and capecitabine. FP‐caused toxicity remains a major clinical issue, comprising up to 30% of reported severe toxicity cases and a mortality rate ranging from 0.5% to 3%.^1^, ^2^

The catabolic activity of FP is well known. A substantial proportion of treatment‐related severe toxicity cases can be explained by the decreased activity of dihydropyrimidine dehydrogenase (DPD), a rate‐limiting enzyme encoded by the *DPYD* gene. Therefore, it is of major importance to assess the DPD activity or identify DPD deficiency before prescribing the first cycle of FP‐based chemotherapy.^3^ Around 3%–10% of patients have partial DPD deficiency and around 0.1%–0.5% complete DPD deficiency.^4^, ^5^

Dihydropyrimidine dehydrogenase deficiency may be identified through two different testing methods: phenotyping or genotyping. A previous study has shown that DPD phenotyping (by measuring uracilemia) could identify cancer patients at risk of severe and life‐threatening FP‐caused toxicity.^6^ This method could help personalize the FP dose in 3%–6% of patients before the first cycle of chemotherapy, although the commonly recommended uracilemia threshold of 16 ng/mL is debated. Moreover, preanalytical steps can substantially interfere with uracilemia results.^7^ Besides phenotyping, genotyping can identify *DPYD* variants unable to catabolize FP. The clinical benefit of genotyping has been proven for two of the four most common and relevant *DPYD* variants (DPYD*2A and c.1679T>G).^8^ Thus, *DPYD* genotyping can also help to personalize the FP dose and improve the FP safety profile. However, the literature data stress the poor concordance between uracilemia and the presence of nonfunctional *DPYD* variants, suggesting these two analytical strategies (genotyping vs. phenotyping) for screening patients at risk of severe FP toxicities are not interchangeable but rather complementary.^9^, ^10^, ^11^

In February 2018, French authorities (ANSM) recommended screening for DPD deficiencies to limit severe FP‐induced toxicities, suggesting a dual approach (phenotyping and genotyping for the four relevant variants: DPYD*2A, c.2846A>T, c.1679T>G, and c.1236G>A). In December 2018, the recommendations were rather in favor of a mandatory uracilemia test and the discontinuation of *DPYD* genotyping. Nevertheless, genotyping, especially using next‐generation sequencing, may help to identify both common and rare nonfunctional *DPYD* variants associated with altered FP catabolism and severe FP toxicity. Thus, we have carried out a real‐life retrospective study to assess (i) the impact of DPD screening on the occurrence of severe toxicity, (ii) the added value of large genotyping using next‐generation sequencing compared with uracilemia alone, and (iii) the concordance between uracilemia and genotyping results.

## 2 METHODS

### 2.1 Population

We conducted a retrospective, multicenter study involving six hospitals in the Hauts‐de‐France region, namely the Oscar Lambret Center, Lille University Hospital, Saint‐Omer Hospital, Tourcoing Hospital, Boulogne‐Sur‐Mer Hospital, and Roubaix Hospital. We reviewed the medical records of all consecutive patients who had undergone fluoropyrimidine‐based (FP‐based) chemotherapy. Patients were eligible if they had received the first cycle of an FP‐based chemotherapy regimen between March 2018 and February 2019, irrespective of the chemotherapy protocol or tumor location. Patients were excluded if they had previously been prescribed FP‐based chemotherapy, had received concomitant hyperthermic intraperitoneal chemotherapy along with 5‐FU, were administered 5‐FU topically, were managed at a different healthcare facility, had undergone genotyping analysis limited to the four most common variants, or were under the age of 18 years. Additionally, patients who had undergone screening but for whom the screening results were unknown before the initiation of the first chemotherapy cycle were also excluded from the analysis. Patient characteristics, details of the chemotherapy protocol employed, and records of early severe toxicities were retrospectively collected using the patients' medical records.

### 2.2 Ethical considerations

The study complies with reference methodology MR004 adopted by the French Data Protection Authority (CNIL), and every participating center was responsible for checking that patients did not object to the use of their clinical data for research purposes. This study was approved by the Institutional Review Board of the Oscar Lambret Center.

### 2.3 Screening procedures

Patients underwent screening for DPD deficiency through three methods: (i) next‐generation sequencing, which aimed to identify DPYD variants (DPYD genotyping), (ii) DPD phenotyping, involving the assessment of uracilemia and the ratio of dihydrouracil (UH2) to uracil (U) in plasma, referred to as the UH2/U ratio (DPD phenotyping) or (iii) a combination of both approaches, known as combined screening. For the DPD phenotyping method, a 5 mL of blood sample was collected in an EDTA tube. The blood required to be centrifuged at 4°C for 10 min at 1500*g*, then decantated and finally frozen in a delay of merely 30 minutes after blood withdrawal. An Ultra‐High Performance Liquid Chromatography Tandem Mass Spectrometric (UPLC‐MS/MS) with Waters Aquity TQD machine was then performed on 1.5 mL plasma sample to calculate in ng/mL uracilemia but also dihydrouracil. *DPYD* genotyping allowed to identify not only patients that carried at least one of the four most common nonfunctional variants (DPYD*2A, DPYD*13, HapB3, and 2864A>T) but also patients carrying rare nonfunctional variants. All screening tests were performed in the toxicology laboratory of Lille University Hospital. The laboratory systematically provided a proposal for an individualized starting dose of FP according to the results based on the pharmacogenetic guidelines^12^ for common variants and the results of in vitro and in silico measurements of DPD activity for rare variants.^13^ For example, in case of heterozygous variant DPYD*2A, DPYD*13 and 2864A>T, the toxicology laboratory recommended to reduce the FP starting dose by 50%. In case of heterozygous HapB3 *DPYD* variant, the toxicology laboratory recommended to reduce the FP starting dose by 25%. In patients with uracilemia above the cutoff of 16 ng/mL without nonfunctional *DPYD* variants, the toxicology laboratory recommended to reduce the FP starting dose by 50%.

### 2.4 Endpoints

We selected clinically relevant endpoints. The primary endpoint of the study was the rate of severe (grade ≥3) FP‐related toxicity occurring between the first and second cycles. Toxicity was assessed using the National Cancer Institute Common Terminology Criteria for Adverse Events version 5.0. The 5‐FU dose reduction in the second chemotherapy cycle was also explored. The definition of 5‐FU dose reduction in our study encompassed any reduction in the dose of 5‐FU administered during the second chemotherapy cycle, regardless of the dose administered during the first cycle. This reduction could vary in magnitude, ranging from a delay in the initiation of the second chemotherapy cycle to a complete elimination of 5‐FU (i.e., a 100% reduction) from the chemotherapy protocol.

### 2.5 Statistics

The characteristics of the study population were described according to the screening strategy. Data were compared using the Mann–Whitney or Kruskal–Wallis test in case of non‐normally distributed quantitative variables and the *χ* ^2^ or Fisher's exact test for qualitative variables. The concordance between genotype and phenotype associated with DPD deficiency was explored using Cohen's kappa.

To study the association between severe early toxicity as the dependent variable and the screening strategy as the explanatory variable, we built multiple logistic regression models adjusting for clinically relevant variables including treatment center, age, sex, curative or palliative management, oral or intravenous chemotherapy, and associated targeted therapy. First, we treated the screening strategy as a four‐modality qualitative variable using the no‐screening strategy as the reference modality versus each of the three other strategies (main model, Model 1). Second, we treated the screening strategy as a binary variable using the no‐screening strategy as the reference modality versus performing a screening, regardless of its type (Model 2). For these two models, all patients were considered (*n* = 551). Third, to explore the added value of large genotyping by next‐generation sequencing compared with uracilemia alone, we studied the associations between severe early toxicity and screening strategy comparing the phenotyping screening strategy as the reference modality to the combined screening (Model 3). For this model, only patients who underwent combined or phenotyping screening were considered (*n* = 440). Similarly, we studied the association between severe early toxicity as the dependent variable and 5‐FU dose reduction as the explanatory variable. Model 4 treated the screening strategy as a four‐modality qualitative variable and was performed considering patients having received at least a second cycle of chemotherapy (*n* = 517). Model 5 compared the phenotyping screening strategy to combined screening and included patients having received at least a second cycle of chemotherapy and who underwent combined or phenotyping screening (*n* = 414). Moreover, we conducted sensitivity analyses to evaluate the robustness of the models. Sensitivity analyses were performed without the exclusion of patients who had been screened but for which the screening results were unknown before the start of the first chemotherapy cycle. Results are presented as odds ratios (ORs) with 95% confidence intervals (CIs).

For all analyses, Stata version 17.0 (Release 17, 2021, StataCorp LLC, College Station, TX) was used.

## 3 RESULTS

### 3.1 Patient characteristics, screening strategies, and treatments

A total of 551 patients were retrospectively included. Patient characteristics, screening strategies, and treatments are summarized in Table 1. The patients were categorized into four groups according to the DPD screening method: 78 (14%) were not screened for DPD deficiency, 33 (6%) were screened by DPD genotyping, 42 (8%) were screened by DPD phenotyping, and 398 (72%) were screened by genotyping and phenotyping (hereafter “combined DPD screening”). The median age at screening was 63 years (range: 26–89), and 214 patients (39%) were women. The most prevalent cancers were tumors of the digestive system (67%), head and neck cancers (20%), and breast cancers (9%). No significant differences among the four groups were observed except for primary tumor sites (*p* = 0.001) a use of targeted therapy (*p* = 0.02) and use of immunotherapy (*p* = 0.01).

**TABLE 1:** Population characteristics, screening strategies and treatment.

	No screening		Combined screening		DPD genotype		DPD phenotype		Total		*p*‐value
	*N* = 78		*N* = 434		*N* = 41		*N* = 44		*N* = 597		
Age at screening (years)	61.5	26–89	63.5	32–87	64.2	40–79	60.7	31–81	63.2	26–89	0.43
Sex											0.26
Male	30	49%	110	60%	48	61%	50	75%	170	61%	
Female	32	41%	174	40%	16	39%	11	25%	233	39%	
Primary sites											0.001*
Upper digestive tract	29	37%	151	35%	11	27%	5	11%	196	33%	
Lower digestive tract	17	22%	159	37%	20	49%	16	36%	212	36%	
Head and neck	14	18%	76	18%	6	15%	20	46%	116	19%	
Gynecological breast	15	19%	37	8%	1	2%	2	5%	55	9%	
Other	3	4%	11	2%	3	7%	1	2%	18	3%	
Management											0.43
Curative‐intent	29	37%	200	46%	21	51%	20	45.5%	270	45%	
Palliative	49	63%	234	54%	20	49%	24	54.5%	327	55%	
Chemotherapy											0.20
5FU IV	70	90%	380	88%	33	80%	42	95.5%	525	88%	
Oral capecitabine	8	10%	54	12%	8	20%	2	4.5%	72	12%	
Bolus (MD = 10)											0.25
No	25	36%	167	45%	14	42%	23	55%	229	45%	
Yes	45	64%	203	55%	19	58%	19	45%	286	55%	
Targeted therapy											0.005
None	64	82%	365	84%	33	81%	32	73%	494	83%	
Bevacizumab	5	6%	28	7%	2	5%	0	–	35	6%	
Cetuximab	5	6%	32	7%	6	15%	12	27%	55	9%	
Other	4	5%	9	2%	0	–	0	–	13	2%	
Immunotherapy – (MD = 5)											0.02
No	75	96%	430	100%	40	100%	44	100%	589	99%	
Yes	3	4%	0	–	0	–	0	–	3	1%	
Result of the screening test											0.29
No deficit—Not at risk of severe toxicity	–	–	393	91%	38	93%	43	98%	474	91%	
Deficit—At risk of severe toxicity	–	–	41	9%	3	7%	1	2%	45	9%	
First chemotherapy dose performed with knowledge of the suspect deficit (*N* = 45)											0.61
No	–	–	8	20%	1	33%	0	–	9	20%	
Yes	–	–	33	80%	2	67%	1	100%	36	80%	
Second cure of chemotherapy											0.46
No	7	9%	28	7%	1	2%	1	2%	37	6%	
Yes	71	91%	406	93%	40	98%	43	98%	560	94%	
Reason not for (*N* = 37)											0.66
Alteration of general condition or progression	2	29%	11	38%	0	–	0	–	13	34%	
Death	1	14%	6	21%	1	100%	1	100%	9	24%	
Toxicity	2	29%	8	28%	0	–	0	–	10	26%	
Other	2	29%	4	14%	0	–	0	–	6	16%	
Modification (*N* = 560)											0.04
No	57	80%	330	81%	32	80%	27	63%	446	80%	
Yes	14	20%	76	19%	8	20%	16	37%	114	20%	

*Note*: Age is described using median [interquartile range, IQR], comparison between groups was performed using a Mann–Whitney test. Qualitative variables are described using number (percentage), comparison between groups was performed using Pearson *χ* ^2^ tests or Fisher's exact test.

* Other cancer, ENT (Ear, Nose, Throat) cancer and gynecological cancer were combined in order to perform Fisher's exact test.

Among the 473 patients with DPD screening, none presented complete DPD deficiency, but 36 patients (8%) presented DPD deficiency (Table 1). This rate was higher in the combined screening group (8% vs. 6% and 2% compared with the DPD genotyping and DPD phenotyping groups, respectively). However, this difference did not reach the level of significance (*p* = 0.45).

In total, 517 patients (94%) received a second cycle of chemotherapy. The second cycle FP dose was significantly more frequently reduced in the DPD phenotyping group compared with the other groups (37% vs. 20%, 20%, and 19% in the no‐screening, combined DPD testing, and DPD genotyping groups, respectively; *p* = 0.08).

### 3.2 Concordance between DPD genotype and phenotype

The concordance between the DPD genotype and phenotype was tested and appeared to be weak with Cohen's kappa of 0.14.

### 3.3 Toxicity in the first and second cycles of chemotherapy

The proportions of patients experiencing severe toxicity events are shown in Table 2. In total, 73 patients (13%) experienced severe toxicity effects between the first and the second chemotherapy cycle, including two patients with grade 5 digestive toxicity, four patients with grade 4 digestive toxicity, and 23 patients with grade 4 hematological toxicity. Severe toxicity occurred more frequently in the no‐screening group (17% vs. 13%, 12%, and 7% in the combined DPD screening, DPD genotyping, and DPD phenotyping groups, respectively). However, this difference was not statistically significant (*p* = 0.56).

**TABLE 2:** Severe toxicity reported.

Severe toxicity reported	No screening		Combined screening		DPD genotype		DPD phenotype		Total		*p*‐value
	*N* = 78		*N* = 434		*N* = 41		*N* = 44		*N* = 597		
All	13	17%	55	13%	5	12%	4	9%	77	13%	0.69
Blood disorder	11	14%	31	7%	3	7%	4	9%	49	8%	0.23
General disorder	3	4%	16	4%	0	–	1	2%	20	3%	0.80
Gastrointestinal disorder	3	4%	26	6%	2	5%	1	2%	32	5%	0.82
Hand foot syndrome	0	–	1	0%	0	–	0	–	1	0%	–

*Note*: Data are *n* (%), comparison between groups was performed using Pearson *χ* ^2^ tests or Fisher's exact test.

### 3.4 Association between screening strategy and severe toxicity events

Multivariate analyses between the occurrence of severe toxicity and clinically relevant variables are presented in Table 3. In the main model, severe toxicity was not significantly associated with the screening strategy. Compared with the no‐screening group, combined DPD screening, DPD genotyping, and DPD phenotyping groups had adjusted ORs of 0.76 (95% CI 0.37–1.54), 0.77 (95% CI 0.22–2.68), and 0.40 (95% CI 0.10–1.58), respectively (*p* = 0.63). In Models 2 and 3, severe toxicity was also not significantly associated with the screening strategy. Model 2 showed that compared with the no‐screening group, DPD screening groups had an adjusted OR of 0.73 (95% CI 0.36–1.48, *p* = 0.39). Likewise, Model 3 showed that compared with DPD phenotyping, combined DPD screening did not, as hypothesized, significantly lower the risk of severe toxicity. On the contrary, the adjusted OR was above 1 (OR 1.83, 95% CI 0.53–6.41, *p* = 0.34), although not significant. The sensitivity analysis confirmed these results (Supplementary data).

**TABLE 3:** Multivariate analysis between severe early toxicity and clinically relevant variables.

Variables	*N* = 597			*N* = 597			*N* = 478		
	Main model			Model 2			Model 3		
	Number event/N	OR	*p*‐value	Number event/N	OR	*p*‐value	Number event/N	OR	*p*‐value
		[95% CI]			[95% CI]			[95% CI]	
Screening strategy			0.65			0.26			0.61
No screening (ref)	13/78	1		13/78	1		–	–	
Combined screening	55/434	0.67 [0.33–1.37]		–	–		55/434	1.34 [0.44–4.11]	
DPD genotype	5/41	0.73 [0.23–2.31]		–	–		–	–	
DPD phenotype	4/44	0.49 [0.14–1.73]		–	–		4/44	1	
Screening strategy (all)		–		64/519	0.66 [0.33–1.35]		–	–	
Sex			0.01			0.007			0.002
Female (ref)	41/233	1		41/233	1		34/185	1	
Male	36/364	0.51 [0.31–0.84]		36/364	0.50 [0.31–0.83]		25/293	0.40 [0.22–0.71]	
Age, years	77/597	0.99 [0.98–1.02]	0.99	77/597	1.00 [0.98–1.02]	0.97	59/478	1.00 [0.98–1.03]	0.75
Stage			0.80			0.80			
Curative (ref)	31/270	1		31/270	1		21/220	1	0.18
Palliative	46/327	1.07 [0.63–1.84]		46/327	1.07 [0.63–1.84]		38/258	1.54 [0.83–2.85]	
Chemotherapy			0.07			0.08			0.03
IV 5FU (ref)	71/525	1		71/525	1		56/422	1	
Oral 5FU	6/72	0.42 [0.16–1.08]			0.43 [0.16–1.10]			0.24 [0.07–0.86]	
Chemotherapy period			0.47			0.49			0.20
03 to 08/2018 (ref)	36/289	1		36/289	1		23/216	1	
09/2018 to 02/2019	41/308	1.20 [0.73–1.99]		41/308	1.19 [0.72–1.97]		36/262	1.46 [0.82–2.61]	
Targeted therapy			0.61			0.63			0.95
No	61/494	1		61/494	1		47/397	1	
Yes	16/103	1.19 [0.62–2.29]		16/103	1.17 [0.61–2.25]		12/81	0.98 [0.46–2.07]	

*Note*: The models are logistic regressions with severe toxicity as the dependent variable. Models are adjust on clinically relevant variables: center, age, gender, curative or palliative management, oral or intravenous chemotherapy, period and associated targeted therapy. In the main model and in Model 2, all patients are considered (*N* = 597). In Model 3, patients who benefit from a combined or a phenotyping screening are considered (*N* = 478).

Abbreviations: 95% CI, 95% confidence interval; OR, odds ratio.

### 3.5 Association between screening strategy and FP dose reduction in the second cycle

Multivariate analyses of the relationships between 5‐FU dose reduction and clinically relevant variables are presented in Table 4. In Model 4, 5‐FU dose reduction was not significantly associated with the screening strategy (*p* = 0.13). Compared with the no‐screening group, the DPD phenotyping group had an adjusted OR of 2.48 (95% CI 0.97–6.32). Model 5 showed that compared with DPD phenotyping, combined DPD screening necessitated a significantly lower frequency of 5‐FU dose reduction with an adjusted odds ratio of 0.43 (95% CI 0.19–0.80, *p* = 0.02). The sensitivity analysis confirmed these results.

**TABLE 4:** Multivariate analysis between 5‐FU dose reduction and clinically relevant variables.

Variables	*N* = 597			*N* = 449		
	Model 4			Model 5		
	Number event/N	OR	*p*‐value	Number event/N	OR	*p*‐value
		[95% CI]			[95% CI]	
Screening strategy			0.07			0.01
No screening (ref)	14/71	1		–	–	
Combined screening	76/406	1.11 [0.55–2.25]		76/406	0.39 [0.19–0.80]	
DPD genotype	8/40	1.07 [0.39–2.92]		–	–	
DPD phenotype	16/43	2.80 [1.09–7.19]		16/43	1	
Screening strategy (all)	–	–		–	–	
Sex			0.74			0.38
Female (ref)	42/217	1		36/171	1	
Male	72/343	0.93 [0.59–1.45]		56/278	0.80 [0.48–1.32]	
Age, years	114/560	1.00 [0.98–1.02]	0.92	92/449	0.99 [0.97–1.01]	0.54
Stage			0.54			0.24
Curative (ref)	52/261	1		45/215	1	
Palliative	62/299	0.86 [0.54–1.37]		47/234	0.73 [0.44–1.23]	
Chemotherapy			0.27			0.26
IV 5FU (ref)	105/489	1		85/394	1	
Oral 5FU	9/71	0.63 [0.28–1.42]		7/55	0.59 [0.24–1.46]	
Targeted therapy			0.045			0.02
No	86/462	1		68/371	1	
Yes	28/98	1.78 [1.01–3.11]		24/78	2.12 [1.13–3.98]	

*Note*: The models are logistic regressions with 5‐FU dose reduction as the dependent variable. Models are adjust on clinically relevant variables: center, age, gender, curative or palliative management, oral or intravenous chemotherapy, period, and associated targeted therapy. In the Model 4, patients who received at least a second cycle of chemotherapy are considered (*N* = 560). In Model 5, patients who received at least a second cycle of chemotherapy and who benefit from a combined or a phenotyping screening are considered (*N* = 449).

Abbreviations: 95% CI, 95% confidence interval; OR, odds ratio.

The use of targeted therapy (mostly anti‐EGFR therapy) was at the threshold of statistical significance in model 4 (OR = 1.74, 95% CI 0.98–3.07, *p* = 0.06) for 5‐FU dose reduction and significant in model 5 (OR = 2.07, 95% CI 1.09–3.90, *p* = 0.03), possibly suggesting an association with 5‐FU dose reduction.

## 4 DISCUSSION

The present study demonstrates that FP regimens remain frequently used in everyday clinical practice, with data collected from 551 patients across six hospitals over a 12‐month period. This retrospective study focussed on the period just prior to the mandatory requirement for DPD screening by French authorities. Among the 551 enrolled patients, no complete DPD deficiency was identified; consistent with the rarity of this condition (for instance, in a large cohort of 5886 patients, Pallet et al. identified only two cases of complete DPD deficiency).^11^ In the genotyping group, we found that 6% of our patients carried nonfunctional DPYD variants, which is in line of prior findings (4% in the report by Pallet et al.^11^ and about 8% in three other studies^1^, ^8^, ^14^). In the phenotyping group, 2% of patients were poor metabolizers, a lower percentage than previously reported (e.g., 16% in the study by Laures et al.^15^ and 7% in Pallet et al.^11^). However, this percentage should be interpreted with caution, given the small size of this group (*n* = 41). In our study, patients with U levels in blood greater than 16 ng/mL among those screened using phenotype testing represented 4% of patients (18 of 440), which aligns with the literature more accurately. Finally, in the combined DPD screening group, we found that approximately 8% of patients were at high risk of PF toxicity. Our findings also confirm the low concordance between phenotyping and genotyping approaches, consistent with previous studies.^9^, ^16^

The systematic *DPYD* deficiency screening remains a topic of debate in the medical oncology community. For instance, screening for DPD deficiency is not mandatory in the United States or Canada, while in other countries, it is recommended by some pharmacology/oncology societies or authorities (e.g., the European Medicine Agency has recommended screening since April 2020)^17^ or is mandatory, as in France or Sweden.^18^ These variations may be surprising, given the widespread use of FP regimens. Moreover, the prevalence of complete deficiency (0.1%, resulting in an absolute FP contraindication) and partial deficiency (8% of patients, requiring dose adjustments) suggests that screening recommendations should be standardized globally.

Two methodological approaches are available: phenotyping and genotyping. In terms of cost, the assessment of uracilemia is affordable and fully reimbursable in France since August 2019 (B120, i.e., €32.40), which is comparable to the cost of a standard biological check‐up before chemotherapy. In comparison, constitutional and complete *DPYD* genotyping by next‐generation sequencing (NGS) costs €882.90 and is not directly reimbursable. Targeted genotyping (to detect only the four main variants) is less expensive, around €110 to €150, and is also not directly reimbursable. Therefore, economic considerations favor uracilemia detection. Furthermore, the uracilemia test is rapid (7–10 days) and identifies poor metabolizers.^6^ However, it requires adherence to pre‐analytical requirements (e.g., transportation on ice),^19^, ^20^ some confounding factors can affect uracilemia (age, liver tests, and glomerular filtration rate)^21^ and the threshold for defining altered metabolism still under discussion.^22^ On the other hand, genotyping identifies one of the 90 *DPYD* variants associated with decreased FP catabolism.^23^ Among the 90 nonfunctional variants, three are more common (DPYD*2A and DPYD*13 in around 1.6% and 0.2% of the general population respectively, and c.2846A>T in around 1.5% of the Caucasian population).^1^, ^14^, ^23^, ^24^ As shown above, this technique is more expensive and time‐consuming. Hence, it is pertinent to assess the value added by genotyping. In the present study, both strategies were combined in the majority of patients.

We aimed to conduct a real‐life study, during the period when DPD screening became mandatory by French health authorities. We intended to analyze how DPD deficiency screening was implemented into current practice, which methods were preferred, and what kind of information these methods generated. Concerning our primary endpoint, severe FP‐related toxicity after the first cycle of treatment, we did not observe any differences based on the strategy used for DPD screening, whether it was phenotyping, genotyping, or a combination of both. It is important to note that our retrospective study lacks sufficient evidence to establish definitive recommendations for the optimal screening strategy.

This study is subject to several limitations. First, it was a retrospective study, which means that some data were either missing or imprecise, particularly regarding the occurrence of toxicity. Additionally, the sample size was relatively small, which restricted our ability to directly compare the different screening strategies effectively. We reported the techniques that were employed, namely uracilemia detection (phenotyping), genotyping, or a combination of both. These analyses were conducted in an ongoing manner, driven by the increasing regional demand, and with the near‐term expectation of a regulatory requirement. Groups corresponding to the various screening strategies were not established prospectively but rather formed after the fact, a characteristic shared with many real‐world studies.

Furthermore, our study has several additional limitations. First, we did not provide precise information regarding the doses of 5‐FU administered during the first and second chemotherapy cycles. Instead, we simply reported whether the 5‐FU dosing was reduced in the second cycle compared to the dose used in the first cycle of chemotherapy. Consequently, due to the small sample size, we were unable to conduct a detailed analysis of the changes in 5‐FU dosing in the second cycle (whether there was a reduction or escalation) for patients who initially had a dose reduction in the first cycle due to suspected DPD deficiency.

Another limitation in our study is the relatively small number of patients who presented with partial DPD deficiency (*n* = 36). This limited sample size makes it challenging for us to statistically demonstrate the superiority of one DPD testing method over another.

Lastly, we had to exclude 46 patients from our study who underwent DPD testing before starting FP chemotherapy but began treatment without waiting for the test results. This may have been due to delays in obtaining these results during the period when systematic screening was being implemented. In such cases, some oncologists may have opted for an arbitrary dose reduction in the first cycle. This situation could potentially introduce bias and affect the validity of the control group (comprising patients who did not undergo screening). Unfortunately, we did not collect data on this aspect of the study.

Furthermore, it is important to acknowledge that using the occurrence of toxicity after the first cycle of chemotherapy as an endpoint can be subject to debate, especially considering that most treatment regimens involve polychemotherapies. Various other factors, such as patient age, comorbidities, overall health status, the specific polychemotherapy protocol used, and co‐medications (e.g., cetuximab), may contribute to the observed effects on toxicity.

One notable finding in our study is the difference in FP dose reduction in the second cycle between the group of patients who underwent phenotype‐only screening and those who received no screening, with an odds ratio of 2.48 [0.97–6.32]. This seemingly unusual result may be attributed to the fact that, during this period when there was a lack of consensus on the recommended screening type, many healthcare practitioners may have chosen to arbitrarily reduce the dose in the first cycle for patients who did not undergo DPD testing. It is worth noting that detailed data on the exact dose of 5‐FU administered during the first cycle were not systematically collected in our study, and this potential bias should be considered when interpreting this result.

## 5 CONCLUSIONS

The present study revealed the widespread adoption of DPD screening strategies in anticipation of recommendations from French health authorities. Concerning our primary outcome, we faced challenges in adequately assessing the added value of this innovative next‐generation sequencing genotyping technique, either alone or in combination with uracilemia, in reducing severe FP‐related toxicities. In fact, none of the methods employed for DPD deficiency screening demonstrated any benefit in terms of reducing the incidence of severe toxicity when compared to an absence of screening. This result was likely influenced by the limited number of patients and other discussed biases.

The current screening tests are primarily effective in identifying patients with complete DPD deficiency, for whom treatment with fluoropyrimidines is strictly contraindicated. Fortunately, complete DPD deficiency is a rare condition, and no cases were identified in this study. Further clinical evaluation is needed to determine the optimal approach to DPD screening, particularly with the utilization of next‐generation sequencing genotyping methods.

## AUTHOR CONTRIBUTIONS

**Côme De Metz:** Conceptualization (equal); writing – original draft (equal); writing – review and editing (equal). **Benjamin Hennart:** Writing – review and editing (supporting). **Estelle Aymes:** Formal analysis (lead); software (lead); visualization (equal); writing – review and editing (equal). **Pierre‐Yves Cren:** Formal analysis (lead); software (lead); writing – original draft (lead); writing – review and editing (lead). **Niels Martignène:** Formal analysis (supporting); software (supporting); writing – review and editing (equal). **nicolas penel:** Writing – original draft (lead); writing – review and editing (lead). **Maël Barthoulot:** Formal analysis (lead); software (lead); writing – original draft (lead); writing – review and editing (lead). **Aurélien Carnot:** Conceptualization (equal); writing – review and editing (equal).

## FUNDING INFORMATION

This research received no external funding.

## CONFLICT OF INTEREST STATEMENT

The authors declare no conflict of interest.

## ETHICS STATEMENT

This study complies with reference methodology MR004 adopted by the French Data Protection Authority (CNIL), and every participating center was responsible for checking that patients did not object to the use of their clinical data for research purposes. This study was approved by the Institutional Review Board of the Oscar Lambret Center.

## PATIENT CONSENT STATEMENT

Patients did not object to the use of their clinical data for research purposes.

## Supporting information




Appendix1

## Data Availability

Requests for access to the data can be submitted to the corresponding author.
