# Supplementary material for: Complete DPYD genotyping combined with dihydropyrimidine dehydrogenase phenotyping to prevent fluoropyrimidine toxicity: A retrospective study
Source: Cancer Med. 2024 Mar 25;13(6):e7066. doi: 10.1002/cam4.7066 (PMC10961597; doi:10.1002/cam4.7066)
Supplement: Supplementary file 1 — Appendix1 [file CAM4-13-e7066-s001.doc]

# Sensitivity analysis

Each model were performed after exclusion of the 46 patients that were screened but for which the screening results were unknown before start of first chemotherapy.

## Main Model

The results of the multivariable logistic regression sensitivity analysis confirmed that the screening strategy was not associated with decrease in early severe toxicities. Compared with no screening patients, combined DPD screening patients, genotyping DPD screening patients and phenotyping DPD screening patients had adjusted odds ratios (ORs) of 0.74 (0.36–1.52) , 0.75 (0.22–2.63) and 0.39 (0.10-1.55), respectively (p=0.60).

Table 17 : Risks of any type of severe toxicity (N=551)

| **Factors** | **Univariate analysis** | | | **Multivariate analysis*** | |
| --- | --- | --- | --- | --- | --- |
|  | **Number event/N** | **OR**  **[95% CI]** | **P-value** | **OR**  **[95% CI]** | **P-value** |
| **Screening strategy** |  |  | 0.55 |  | 0.63 |
| No screening (ref) | 13/78 | 1 |  | 1 |  |
| Combined screening | 53/398 | 0.77 [0.40-1.49] |  | 0.76 [0.37-1.54] |  |
| Genotyping only | 4/33 | 0.69 [0.21-2.30] |  | 0.77 [0.22-2.68] |  |
| Phenotyping only | 3/42 | 0.38 [0.10-1.43] |  | 0.40 [0.10-1.58] |  |
| **Sex** |  |  | 0.01 |  | 0.64 |
| Female (ref) | 39/214 | 1 |  | 1 |  |
| Male | 34/337 | 0.50 [0.31-0.83] |  | 0.52 [0.31-0.86] |  |
| **Age, years** | 73/551 | 0.99 [0.97-1.01] | 0.37 | 0.99 [0.97-1.02] | 0.65 |
| **Stage** |  |  | 0.41 |  | 0.82 |
| Curative (ref) | 30/251 | 1 |  | 1 |  |
| Palliative | 43/300 | 1.23 [0.75-2.03] |  | 1.07 [0.61-1.86] |  |
| **Chemotherapy** |  |  | 0.27 |  | 0.11 |
| IV 5FU (ref) | 67/484 | 1 |  | 1 |  |
| Oral 5FU | 6/67 | 0.61 [0.25-1.47] |  | 0.45 [0.17-1.18] |  |
| **Targeted therapy** |  |  | 0.54 |  | 0.75 |
| No | 58/452 | 1 |  | 1 |  |
| Yes | 15/99 | 1.21 [0.66-2.24] |  | 1.12 [0.57-2.18] |  |

*The results are presented for each variable adjusted on the other variables.

N : total population

95%CI = 95% confidence interval

## Model 2

| **Variables** | **N=551** | | |
| --- | --- | --- | --- |
|  |  | **Model 2 (SE)** |  |
|  | **Number event/N** | **OR** | **P-value** |
|  |  | **[95% CI]** |  |
| **Screening strategy** |  |  | 0.36 |
| No screening (ref) | 13/78 | 1 |  |
| Combined screening | / | / |  |
| DPD genotype | / | / |  |
| DPD phenotype | / | / |  |
| Screening strategy (all) | 60/473 | 0.71 [0.35-1.47] |  |

## Model 3

| **Variables** | **N=551** | | |
| --- | --- | --- | --- |
|  |  | **Model 2 (SE)** |  |
|  | **Number event/N** | **OR** | **P-value** |
|  |  | **[95% CI]** |  |
| **Screening strategy** |  |  | 0.33 |
| No screening (ref) | / |  |  |
| Combined screening | 53/398 | 1.87 [0.54-6.52] |  |
| DPD genotype | / |  |  |
| DPD phenotype | 3/42 | 1 |  |
|  |  |  |  |

## Model 4

The results of the multivariable logistic regression sensitivity analysis confirmed that the screening strategy was not associated with 5FU dose reduction.

Table 18 : Risks of modification of second chemotherapy (N=517)

| **Factors** | **Univariate analysis** | | | **Multivariate analysis*** | |
| --- | --- | --- | --- | --- | --- |
|  | **Number event/N** | **OR**  **[95% CI]** | **P-value** | **OR**  **[95% CI]** | **P-value** |
| **Screening strategy** |  |  | 0.10 |  | 0.13 |
| No screening (ref) | 14/71 | 1 |  | 1 |  |
| Combined screening | 73/373 | 0.99 [0.52-1.88] |  | 1.09 [0.54-2.21] |  |
| Genotyping only | 6/32 | 0.94 [0.32-2.72] |  | 0.99 [0.33-2.95] |  |
| Phenotyping only | 15/41 | 2.35 [0.99-5.57] |  | 2.53 [0.98-6.58] |  |
| **Sex** |  |  | 0.86 |  | 0.64 |
| Female (ref) | 41/200 | 1 |  | 1 |  |
| Male | 67/317 | 1.04 [0.67-1.61] |  | 0.89 [0.56-1.42] |  |
| **Age, years** |  | 1.00 [0.98-1.02] | 0.77 | 1.00 [0.98-1.02] | 0.79 |
| **Stage** |  |  | 0.70 |  | 0.65 |
| Curative (ref) | 49/243 | 1 |  | 1 |  |
| Palliative | 59/274 | 1.09 [0.71-1.66] |  | 0.89 [0.56-1.44] |  |
| **Chemotherapy** |  |  | 0.13 |  | 0.30 |
| IV 5FU (ref) | 99/451 | 1 |  | 1 |  |
| Oral 5FU | 9/66 | 0.56 [0.27-1.17] |  | 0.65 [0.29-1.47] |  |
| **Targeted therapy** |  |  | 0.04 |  | 0.07 |
| No | 81/423 | 1 |  | 1 |  |
| Yes | 27/94 | 1.70 [1.02-2.83] |  | 1.73 [0.98-3.06] |  |

*The results are presented for each variable adjusted on the other variables.

N : total population

95%CI = 95% confidence interval

## Model 5

| **Variables** | **N=551** | | |
| --- | --- | --- | --- |
|  |  | **Model 2 (SE)** |  |
|  | **Number event/N** | **OR** | **P-value** |
|  |  | **[95% CI]** |  |
| **Screening strategy** |  |  | 0.01 |
| No screening (ref) |  |  |  |
| Combined screening | 73/373 | 0.43 [0.21-0.88] |  |
| DPD genotype | / |  |  |
| DPD phenotype | 15/41 | 1 |  |
|  |  |  |  |
